# CTK-like syndrome: Corneal opacity and flattening following sequential intracorneal ring implantation and corneal cross-linking

**DOI:** 10.1016/j.ajoc.2024.102196

**Published:** 2024-10-25

**Authors:** Bruno M. Fontes, Ricardo M. Nosé, Farhad Hafezi, Emilio A. Torres-Netto

**Affiliations:** a Department of Ophthalmology and Visual Sciences, Paulista School of Medicine, Federal University of São Paulo, São Paulo, Brazil; b Centro de Microcirurgia & Diagnóstico, Rio de Janeiro, Brazil; c Eye Clinic, São Paulo, Brazil; d ELZA Institute, Zurich, Switzerland; e Ocular Cell Biology Group, Center for Applied Biotechnology and Molecular Medicine, University of Zurich, Switzerland; f Faculty of Medicine, University of Geneva, Geneva, Switzerland; g School of Ophthalmology and Optometry, Wenzhou Medical University, Wenzhou, Zhejiang, China; h Department of Ophthalmology at NYU Grossman School of Medicine, New York, NY, USA

**Keywords:** Central toxic keratopathy, Cornea, Corneal flattening, Intracorneal ring, Corneal cross-linking, Keratoconus

## Abstract

**Purpose:**

This is a case report of a 31-year-old male patient presenting progressive and markedly asymmetric keratoconus treated with sequential intracorneal ring segment (ICRS) implantation followed by accelerated corneal cross-linking (CXL).

**Observations:**

The follow-up after the last procedure revealed a thin, opacified cornea with an unexpected massive flattening of up to 20.3 D. The central flattening attributed to the individual effect of CXL (post-ICRS-implant *vs.* post-CXL) was 19.1 D.

**Conclusions and Importance:**

This original case reports how CXL followed by ICRS implant may result in an early and extreme corneal remodeling. Moreover, such an unusual combination of extreme corneal flattening, thinning, and opacification may imitate a clinical manifestation of central toxic keratopathy and suggests that eyes with ICRS implantation must be followed closely if CXL is performed sequentially after.

## 1 Introduction

With an estimated prevalence between 0.05 and 4.79 %,^1^^,^^2^ keratoconus (KC) is a corneal ectatic disease that usually presents as an asymmetric bilateral protrusion with focal thinning and abnormal curvature, potentially leading to variable and reduced visual acuity.^3^ According to the clinical stage in which the disease is diagnosed, the treatment may involve distinct approaches: correcting refractive errors as an attempt to improve vision (by spectacles, contact lenses, or corneal surgeries), and/or prevention of ectasia progression. Progression is identified by a ≥1 D increase in Kmax, ≥20 μm thinning at the corneal thinnest point, or >15 μm increase in posterior elevation. Regular follow-up every 3–6 months with corneal topography/tomography, and pachymetry is critical for timely detection and intervention, particularly in younger patients.

Defining corneal ectasia progression remains challenging, as no consistent objective criteria exist, as noted by the 2015 Global Consensus on Keratoconus.^3^ Progression is typically characterized by focal thinning and steepening of the anterior and/or posterior corneal surfaces, with Kmax, representing maximum anterior curvature, being the most commonly used parameter. However, Kmax has limitations, especially in detecting early or subclinical disease. The Consensus emphasized the need for at least two indicators of progression, such as anterior/posterior steepening or corneal thinning beyond measurement variability.^3^ To overcome these limitations, the Belin ABCD classification was developed, incorporating anterior and posterior curvature, corneal thickness, and distance visual acuity. This system, available on the Scheimpflug imaging system [Pentacam, (Oculus GmbH, Wetzlar, Germany)], allows for earlier detection and more comprehensive monitoring of keratoconus, facilitating timely intervention.^4^ Looking ahead, the 2nd Global Consensus is planned for publication in 2025. As of this writing, it is in the final stages of expert evaluation, promising further advancements in the diagnosis and management of ectatic diseases.

Until 2003, no therapy was available to arrest the progressive and abnormal bulging of the cornea. Currently, corneal cross-linking (CXL) has been successfully applied in clinics for more than 15 years and has become the standard treatment for progressive forms of keratoconus.

It has been shown in multiple studies that CXL successfully stops keratoconus^5^ progression and arrests post-surgical corneal ectasia.^6^ In addition to ectasia stabilization, CXL long-term results confirm the ability also to improve curvature and visual acuity in some cases.^7^ A corneal remodeling that causes mild or moderate flattening is observed in most patients.^7^

Originally reported after excimer laser corneal ablation, central toxic keratopathy (CTK) is a rare, idiopathic, non-inflammatory condition presenting central corneal opacification, thinning, and flattening that leads to variable degrees of hyperopic shift. This condition usually appears in the recent postoperative period, is unresponsive to steroid therapy, and tends to be self-limited, including late haze resolution, which typically results in a ‘watchful waiting’ strategy being employed.^8^

We report a case of massive remodeling effect following sequential intracorneal ring segment (ICRS) implantation and CXL. To our knowledge, this case report describes not only the greatest remodeling effect involving CXL in the literature, with extreme central flattening of 20.3 D, but also a CTK-like syndrome reported following CXL treatment.

## 2 Case report

**Fig. 1:**
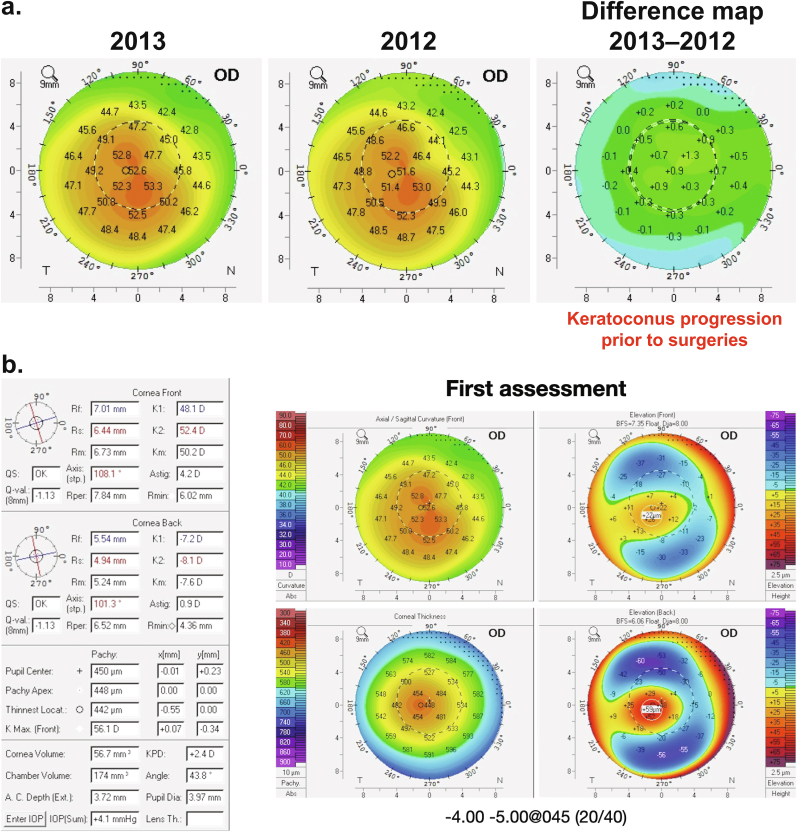
a. Difference map of the anterior corneal curvature of the right eye showing the progressive character of keratoconus before all procedures b. Preoperative corneal tomography at the first assessment, as evidenced by corneal thinning, anterior curvature steepening, and increased posterior elevation.

A 31-year-old male, with markedly asymmetric KC, presented at the initial consultation with progressive unilateral disease, complaining of low visual acuity in his right eye. At this point, progression has been confirmed clinically by unstable refraction and by a Scheimpflug corneal topographer/tomographer (Pentacam, Oculus Optikgeräte, Wetzlar, Germany): differential map showed steepening of 1.3 D in anterior corneal curvature in past 10 months of follow-up (Fig. 1a), and anterior corneal maximum keratometry (Kmax) had increased 1.7 D (from 55.6 to 57.3 D) over a 22-month period. In our first assessment, the patient had a best-corrected visual acuity (BCVA) of 20/40 (subjective refraction: −4.00 sph −5.00 cyl at 45°) on the right eye (Fig. 1b), and 20/20 (subjective refraction: −0.75 sph −0.50 cyl at 60°) on the left eye, leading to high anisometropia. The slit lamp examination revealed a clear cornea with subtle central protrusion and thinning; no opacities or irregularities were observed biomicroscopically. Also, the patient was highly intolerant to contact lens use, even if reaching 20/20 visual acuity with a rigid gas permeable contact lens test.

The rationale for the elected treatment was as follows. Initially, we have chosen to have a surgical procedure that could potentially not only improve the corneal shape due to remodeling, but also improve anisometropia and deliver to the patient a better corrected visual acuity with spectacles. Therefore, the initial approach was the implantation of two segments of ICRS. Then, to sustain the results achieved with ICRS implantation and prevent future visual loss, CXL was performed 3 months later.

**Fig. 2:**
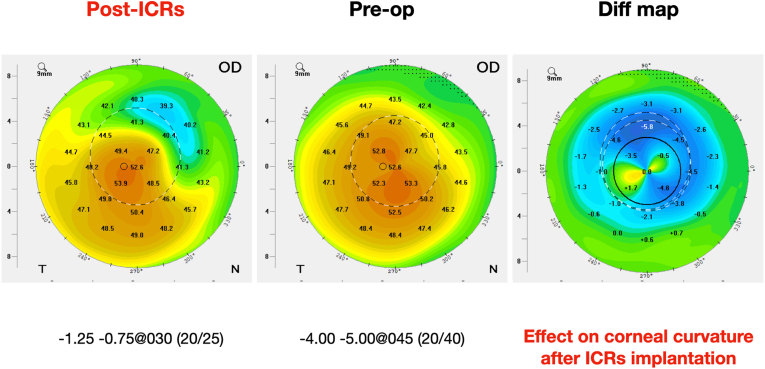
Difference map showing 1-month follow-up cornea remodeling after two intracorneal ring segments implantation.

Intrastromal tunnels for ICRs' implantation were performed using a femtosecond laser (FS200 WaveLight, Alcon, Fort Worth, TX, USA). Two identical segments of ICRs (Ferrara Ophthalmics, Belo Horizonte, Brazil), each with 200 μm of thickness and 140° arc length, were implanted opposite each other following the manufacturer's nomogram, without any intraoperative complications. The ICRS was planned to be implanted at 75 % of the depth at the thinnest point, specifically targeting a planned depth of 330 μm. The postoperative follow-up was unremarkable, with improved BCVA and patient satisfaction. The BCVA with a refraction of −1.25 sph −0.75 cyl at 30° was 20/25, and Scheimpflug images were recorded (Fig. 2).

Three months after the ICR segment implantation, epithelium-off accelerated CXL treatment (9 mW/cm^2^ for 10 minutes, total fluence 5.4 J/cm^2^) was performed using a UVA-light source (Peschke® CCL-365 Vario, Hüenberg, Switzerland). A standard 0.1 % riboflavin solution without dextran and 1.1 % hydroxypropyl methylcellulose (Peschke M®, Hüenberg, Switzerland) was chosen based on central corneal thickness (stromal thickness around 400 μm) and used in the procedure. Corneal endothelial cell count given by specular microscopy was not performed prior to the procedure.

Postoperative eye drops prescription consisted of antibiotic (gatifloxacin 5 mg/ml bid) until complete corneal epithelialization, steroids (prednisolone acetate 1.2 mg/ml) with a staggered 8-week gradual reduction regimen - qid for 2 weeks, tid for 2 weeks, bid for 2 weeks and once a day for additional 2 weeks - and preservatives-free artificial tears (sodium hyaluronate 0.15 mg/ml prn). A bandage contact lens was placed at the end of the surgery and remained on the ocular surface until the epithelium was completed.

The post-CXL follow-up was unremarkable, with complete corneal epithelization occurring 7 days after the procedure, when a demarcation line was clearly visible on the anterior corneal stroma.

**Fig. 3:**
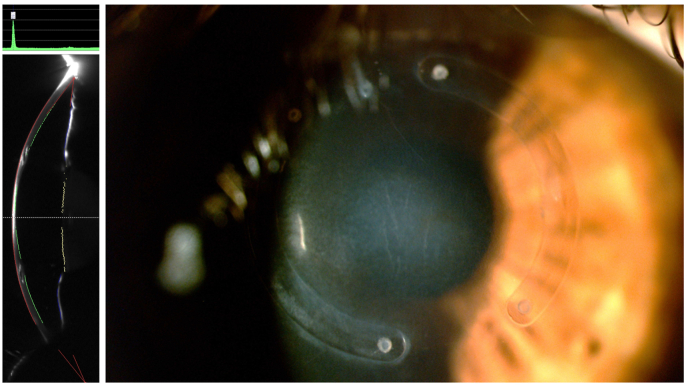
Scheimpflug imaging (left) shows the position of the ICRS and central corneal opacity associated with corneal thinning. Slit lamp corneal imaging (right) displays the equivalent clinical characteristics.

**Fig. 4:**
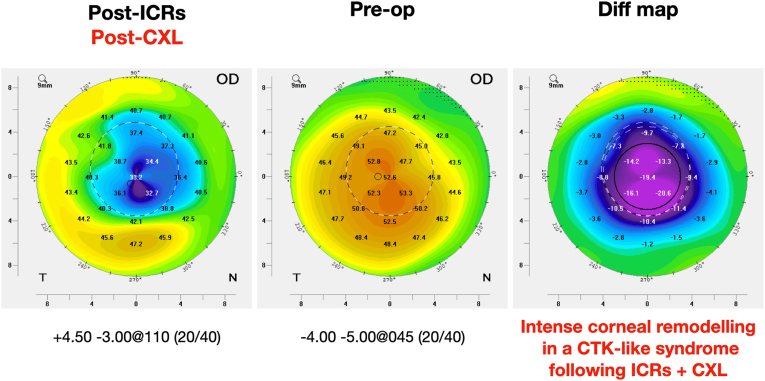
Scheimpflug image comparing the post-ICRS implantation and post-CXL (left) with the preoperative (center) map. The differential map shows the intense corneal flattening of up to 20.6 D after both procedures (right).

Three weeks after CXL, a faint demarcation line at the anterior corneal stroma was visible, and best-corrected visual acuity (BCVA) was 20/50 (−1.00 -1.00 × 130°). The patient missed scheduled monthly appointments and returned four months after the CXL procedure complaining of worsening visual acuity. Dynamic refraction revealed a high hyperopic shift (+4.50 sph −3.00 cyl at 110°) and BCVA of 20/40. Scheimpflug images and slit-lamp examination showed central cornea thinning and opacity confined to the space between the two (well-positioned) ICR segments (Fig. 3). Tomographic images of the cornea revealed an extreme central flattening of up to 20.6 D compared to the same maps before both procedures (Fig. 4). The differential map comparing Scheimpflug images post-ICR-implant *vs.* post-CXL (therefore translating flattening caused by CXL procedure alone) showed a central flattening of up to 19.1 D. Moreover, a clinically relevant corneal thinning could also be noticed at this visit. Although we are aware that corneal opacities may interfere with pachymetric measurements when using Scheimpflug imaging technology, the 203 μm measured central thinning was compatible with the ultrasonic pachymetry (DGH 555 Pachette-3, DGH Technology, Exton, USA) and the clinical examination. Specular microscopy for endothelial evaluation was performed and revealed an endothelial layer with a 2.753 cell count at the center, and normal morphology. Notably, the patient had no systemic or ocular diseases that could have contributed to the postoperative complications. Also, there was no ocular inflammation that could have led to thinning, opacification, or flattening of the cornea. Likewise, no signs of stromal immune or infectious keratitis were noted.

After nine months of CXL follow-up, the hyperopic shift decreased (+1.00 sph −2.00 cyl at 105°), BCVA was 20/30, and haze though still present had reduced. The last Scheimpflug image exam was performed 13 months after the CXL: although with a discrete central steepening of 2.5 D, the cornea still had persistent flattening, with a central keratometry of 35.8 D. The patient has reported a significant improvement in vision over time and expressed satisfaction with the use of spectacles. In light of these findings, the management plan has been adjusted to optimize the ocular surface, which includes the implementation of preservative-free artificial tears and topical cyclosporine to enhance overall ocular health. No surgical intervention was deemed necessary, and ongoing monitoring will be conducted to assess further improvements and explore future treatment options as needed.

## 3 Discussion

This case reports an extreme corneal flattening, stromal thinning, and opacification following a combined sequential ICRS implantation and corneal cross-linking procedure. This report highlights a central toxic keratopathy-like syndrome triggered by the combination of intracorneal ring implantation and corneal cross-linking procedures.

ICR implantation with customized surgical planning can reshape the cornea and partially correct refractive errors. Peripheral tissue addition leads to variable degrees of flattening of the central corneal curvature. The ICR remodeling effect depends on its arc length, radius of curvature, and depth of corneal implantation.^9^^,^^10^ Corneal tunnels can be created either mechanically or by using a femtosecond laser. This technique usually improves the visual acuity of the patient and may be performed before, simultaneously, or after CXL.^3^^,^^10^, ^11^, ^12^, ^13^, ^14^ Some complications after ICRS implantation have been described. In most studies, these include migration, ring extrusion, corneal thinning, corneal melting, and some type of infective keratitis, and these complications together with glare, halos, fluctuating vision, neovascularization, foreign body sensation, or pain represented most of the causes.^15^

The sequencing of surgical interventions in the management of keratoconus remains a topic of considerable controversy in the literature. At the time, the surgeon aimed to facilitate optimal corneal shape improvement before undergoing corneal cross-linking (CXL). Additionally, the surgeon had observed cases of ring extrusion when ICRS implantation was conducted simultaneously with CXL. Therefore, by opting for the sequential approach, the aim was to minimize these risks and enhance the overall effectiveness of the treatment.

Supporting the adopted perspective, El Awady et al. evaluated the outcomes of collagen cross-linking in keratoconus eyes with intracorneal ring implantation.^16^ Their conclusions indicated that collagen CXL had an additive effect after ring implantation and may be considered an enhancement or stabilizing procedure. A more recent study however has shown that simultaneous ICRS implantation and CXL may provide superior outcomes compared to staged techniques.^17^ The initial search identified 120 related articles and, notably, simultaneous surgery demonstrated superior results than the CXL-first technique, as well as superior outcomes to both CXL-first and ICRS-first techniques concerning steep-K.^17^ As noted, the literature differs, and many surgeons worldwide have varying experiences regarding the optimal sequencing of these procedures, which highlights the need for further research to refine best practices in the management of keratoconus.

CXL is used to improve corneal stiffness and arrest the progression of ectasia. The Dresden protocol, epithelium-off CXL with a total fluence of 5.4 J/cm^2^ and irradiation of 30 minutes with 3 mW/cm^2^, certainly has the greatest body of evidence in CXL technology. Due to changes in tissue properties after CXL, mild or moderate flattening is expected in most patients.^7^ However, intense remodeling may occasionally occur: in a series of over 1000 treatments with CXL, Hafezi et al. reported a massive corneal remodeling in only three of the cases, with a maximum keratometric regression of 9.5 D.^18^ The authors reported little improvement of deep stromal haze after one year, and suggested that such changes might be permanent.^18^ Haze after CXL is a well-documented phenomenon, is typically mild and transient, resulting from keratocyte loss in the corneal stroma.^3^^,^^19^, ^20^, ^21^ The haze generally begins to decrease by the third month postoperatively as the cornea undergoes remodeling and keratocyte repopulation, often resolving by 6–12 months with concurrent improvement in visual acuity. This transient haze is usually paracentral and does not significantly affect visual outcomes, distinguishing it from late-onset permanent scarring, which may be associated with other factors rather than CXL itself.^19^, ^20^, ^21^, ^22^, ^23^, ^24^, ^25^ It is worth noting that, unlike with PRK, Mitomycin C application after CXL increases corneal haze.^26^

Although clinically effective, accelerated protocols of 9 mW/cm^2^ for 10 minutes already show a significant decrease in the stiffening effect.^27^ While this may be considered a disadvantage, accelerated treatments could potentially reduce certain exacerbated responses of the Dresden protocol, while still maintaining reasonable biomechanical clinical stability, which can be confirmed by clinical stability over a long follow-up.^28^ Curiously, unlike most massive remodeling cases described so far, ours shows an intense remodeling occurring after an accelerated CXL protocol.

Both ICR and CXL procedures are expected to cause variable degrees of corneal flattening. Furthermore, a combination of treatments - even in sequential surgeries - may potentially increase their individual flattening effects. Specifically in this case report, however, the unusual combination of extreme corneal flattening, thinning, and opacification is compatible with a clinical manifestation of CTK.

Originally reported after excimer laser corneal ablation, CTK is a rare, idiopathic, non-inflammatory condition presenting central corneal opacification, thinning, and flattening that leads to variable degrees of hyperopic shift. This condition usually appears in the recent postoperative period, and tends to be self-limited, including late haze resolution.^8^ Even though our case has not been followed by excimer laser ablation, it presents with all patterns to those described as CTK. While CTK typically manifests within the first week postoperatively, the delayed onset of a CTK-like syndrome observed in our case series, occurring around one-month post-CXL, may be due to procedural factors specific to CXL. Unlike refractive laser surgeries, CXL involves prolonged UV exposure and can lead to delayed corneal remodeling and keratocyte apoptosis, potentially extending the timeline for clinical manifestation of central corneal opacification. This delayed presentation might be attributed to variations in riboflavin penetration, UV fluence, or corneal thickness changes during the CXL procedure, which could contribute to a protracted inflammatory response or delayed onset of keratocyte loss, ultimately resembling CTK in its clinical course.

Still elusive, CTK is possibly triggered by toxins from glove powder, povidone-iodine solutions, marking pens, or an immune-mediated reaction.^29^ Sonmez et al. hypothesized that the tissue loss is caused by keratocyte apoptosis, resulting in thinning and hyperopic shift secondary to photoactivation by ultraviolet laser energy.^8^ In the present case report, CTK-like occurrence could be secondary to any of these causes; and we speculate that it could also be secondary to a reaction between the acrylic from ICRS, riboflavin, or ultraviolet light exposure (or a combination of these factors) during the CXL procedure. Noticeably, extreme corneal changes were seen within the central area of implantation and action of the ICRS (central cornea), and not at the mid-periphery. There are further potential options for the management of the CTK-like phenotype displayed. Although generally contraindicated during the acute phase, could be cautiously reintroduced to reduce inflammation if significant haze remains. To address the residual visual symptoms, the fitting of specialty contact lenses, such as scleral lenses, may provide both visual rehabilitation and symptomatic relief by masking irregular astigmatism and optimizing visual acuity.

## 4 Conclusions

In summary, this case report of extreme corneal remodeling - after the combined sequential procedure of intrastromal ring implantation and CXL - describes significant corneal remodeling and a CTK-like syndrome following CXL treatment. Although this case does not confirm the etiological cause, it suggests that eyes with intracorneal ring segment implantation should be monitored more closely if cross-linking is performed sequentially afterward. Furthermore, it might also be considered that CXL could be performed prior to the ring implantation surgery. Lastly, the advent of Femto-CAIRS (femtosecond laser–cut corneal allogenic intrastromal ring segments) may bring new perspectives in this regard, warranting further exploration and study.

## CRediT authorship contribution statement

**Bruno M. Fontes:** Writing – review & editing, Writing – original draft, Visualization, Validation, Supervision, Project administration, Methodology, Investigation, Data curation, Conceptualization. **Ricardo M. Nosé:** Writing – review & editing, Writing – original draft, Validation, Supervision, Methodology, Investigation, Data curation, Conceptualization. **Farhad Hafezi:** Writing – review & editing, Writing – original draft, Supervision. **Emilio A. Torres-Netto:** Writing – review & editing, Writing – original draft, Validation, Supervision, Formal analysis.

## Patient consent

Written informed consent was obtained from the patient for the publication of this case report. All procedures were performed in accordance with the tenets of the Declaration of Helsinki and complied with the Health Insurance Portability and Accountability Act of 1996.

## Declaration

After conducting a literature review on April 1, 2024, utilizing PubMed, Google Scholar, and ResearchGate using the keywords “keratoconus,” “corneal cross-linking,” “intracorneal ring segments,” “corneal ectasia,” “corneal remodeling,” and ‘central toxic keratopathy,” we did not find any prior reports documenting the association between combined sequential procedures of intracorneal ring segment implantation and corneal cross-linking with the development of central toxic keratopathy-like syndrome.

## Authorship

All authors attest that they meet the current ICMJE criteria for authorship.

## Financial Support

RMN (none), EATN (none), BMF (none), FH (none).

## Declaration of competing interest

The authors declare the following financial interests/personal relationships which may be considered as potential competing interests: The authors have no conflict of interest.
